# Phase and wave dependent analysis of health expenditure efficiency: A sample of OECD evidence

**DOI:** 10.3389/fpubh.2023.1125975

**Published:** 2023-03-17

**Authors:** Elif Boduroglu, Kazim Baris Atici, Tolga Omay

**Affiliations:** ^1^Business Department, Atilim University, Ankara, Türkiye; ^2^Department of Business Administration, Hacettepe University, Ankara, Türkiye; ^3^Economics Department, Atilim University, Ankara, Türkiye

**Keywords:** COVID-19 daily cases, OECD, health expenditure efficiency, wave and phase dependency, cumulative Fourier transform, unit root testing

## Abstract

**Introduction:**

Health expenditures are a factor that reflects the government's public health policy and contributes to the protection of national health. Therefore, this study focuses on measuring the effectiveness of health expenditures in order to evaluate and improve the public health system and policy during the pandemic period.

**Method:**

In order to examine the effectiveness of health expenditures, the behaviors of the pandemic process were analyzed in two stages. The number of daily cases is analyzed in the first stage by dividing it into waves and phases according to the transmission coefficient (R). For this classification, the discrete cumulative Fourier function estimation is used. In the second stage, the unit root test method was used to estimate the stationarity of the number of cases in order to examine whether the countries made effective health expenditures according to waves and phases. The series being stationary indicates that the cases are predictable and that health expenditure is efficient. Data consists of daily cases from February 2020 to November 2021 for 5 OECD countries.

**Conclusion:**

The general results are shown that cases cannot be predicted, especially in the first stage of the pandemic. In the relaxation phase and at the beginning of the second wave, the countries that were seriously affected by the epidemic started to control the number of cas es by taking adequate measures, thus increasing the efficiency of their health systems. The common feature of all the countries we examined is that phase 1, which represents the beginning of the waves, is not stationary. After the waves fade, it can be concluded that the stationary number of health cases cannot be sustainable in preventing new waves' formation. It is seen that countries cannot make effective health expenditures for each wave and stage. According to these findings, the periods in which countries made effective health expenditures during the pandemic are shown.

**Discussion:**

The study aims to help countries make effective short- and long-term decisions about pandemics. The research provides a view of the effectiveness of health expenditures on the number of cases per day in 5 OECD countries during the COVID-19 Pandemic.

## 1. Introduction

Increasing the effectiveness of health systems during pandemics that leave severe problems in economic and social welfare at the global level will increase the level of resistance against the health shocks that countries have to manage (1). It has therefore led to an examination of the capacities and capabilities of national economies worldwide to prevent, detect, and rapidly respond to the emergence of infectious diseases and other acute forms of public health hazards. Health systems that can respond effectively to such health threats also have a significant advantage in reducing their adverse health, social and economic consequences (2).

The effectiveness of health expenditures is the evaluation of expenditures made by the health system by considering factors such as efficiency, usefulness, and quality. Effective health expenditures aim to be achieved with the minimum cost to provide the highest possible quality of service (3). During epidemics, the importance of health expenditures increases to provide the necessary tools and services to contain the epidemic and prevent the spread of the disease. In addition, health expenditures can increase the capacity to protect and treat the diseases caused by the epidemic and allow society to respond healthily (4). The effectiveness of health expenditures during epidemic periods is significant in preventing the spread of the disease and maintaining a healthy society (5).

For this reason, there are many studies on the effectiveness of health expenditures. In the literature, studies on the effectiveness of health expenditures have been examined with parametric and non-parametric methods. Among non-parametric methods, Data Envelopment Analyse (DEA), free disposal hull (FDH) technique and Malmquist efficiency index were frequently used; parametric methods are OLS, COLS, stochastic frontier approach (SFA), correlation and regression analysis, tobit model, global generalized directional distance function, spatial Durbin model, panel models, econometric models such as unit root tests (1, 6–13). From all this literature, we evaluated that there is no consensus on the theoretical or statistical criteria that should be explicitly used to conduct empirical analyses with short- or long-term data to measure the effectiveness of health expenditures (14). Each method has its advantages and disadvantages, and which is most appropriate may vary depending on the problems and objectives being measured.

Unlike the methods used in the literature, this study presents an indirect test method to measure the efficiency of health expenditure or investment during the COVID-19 period. We derived the equation to indirectly test health expenditure efficiency based on the statistical structure of the series of COVID-19 cases and proved this hypothesis in the proceeding sections. This methodological procedure offers a different approach to testing health expenditure efficiency and is a candidate to contribute to this literature. A new constraint is imposed on the Fourier ADF test using wave structure. Therefore, we have proposed a new approach to the cumulative Fourier ADF tests. Therefore, the results of this study show the efficiency of health expenditure during the COVID-19 epidemic.

Examining the pandemic process from February 2020 to November 2021, this study analyses new daily cases using the Cumulative Fourier function. The study split the country's case numbers into waves and phases according to the contagion coefficient (R). Then, it applies a unit root test to investigate the stationarity of the phases. The results show the effectiveness of health expenditures in waves and phases where case numbers are stationary. The results show that the onset of waves (Phase 1) is unpredictable and a unit root process. However, as expected, the daily cases process is becoming predictable, resulting from stationarity. Therefore, we are in a position to determine the health expenditure efficiency by using only daily cases. This computationally easy result is emerging from our proposed theoretical foundation proposition 1. In proposition one, we have shown that we can test whether the health expenditure efficiency can be checked from the daily COVID cases predictability.

The results of this study can help determine the direction of studies to increase the health expenditure efficiency of countries. In addition, this method does not have to be used only for testing the health expenditure efficiency of the COVID-19 outbreak. It may also be a suitable approach for future epidemics or diseases. At the same time, the applicability of these methods to evaluate productive investments in other sectors, such as the energy, agriculture, or tourism sectors, can be explored.

In conclusion, this study offers a new and unique method to test whether health expenditures are efficient or not. This method uses a unit root test that considers the phase and wave structure. Thus, it shows that the unit root test indirectly measures health expenditures' efficiency using the daily COVID cases. The study points out the importance of efficient use of health expenditures and contributes to previous studies.

## 2. Data and methodology

### 2.1. Data

The effectiveness of health systems in OECD countries is analyzed based on data presented in the Systematic dataset of the COVID-19 policy Report published by Oxford University (2022). Country COVID-19 data are taken from the public website of The Oxford COVID-19 Government Response Tracker (OxCGRT). The start dates of the new case data used in the study differ according to the countries. As seen in Table 1, the initial dates of the data start according to the case reporting date of the countries in February 2020. The data expiry date also differs from country to country. The expiry date of the data ends in November 2021. This end-day differs for some reason, such as the start of the Omicron variant, the relaxation of the stiffness index measures on a country-by-country basis, or changes in testing policies. For the sample, Australia (AUS), Canada (CAN), Germany (DEU), France (FRA), and America (USA) are countries within the group of advanced economies, among the top 10 in the human development index (2020–2021), and relatively high populations among OECD countries have been selected. Thus, it is aimed to create a homogeneous sample by considering developed countries in the study.

**Table 1:** Data statistics.

**Country**	**Date range**	**Observations**
AUS	26.01.2020–2.11.2021	646
CAN	26.01.2020–3.11.2021	647
DEU	27.01.2020–4.11.2021	647
FRA	24.01.2020–30.09.2021	615
USA	22.01.2020–10.11.2021	658

### 2.2. Hypothesis and the theoretical fundamentals

Daily COVID-19 case data was used to indirectly measure the effectiveness of health expenditure. There are studies in the literature in which many different methods use health expenditure data. In the literature, inpatient beds, medical technology indicators, and health employment are often examined to measure the effectiveness of health expenditures, while variables of the human development index (Life expectancy, infant mortality, infection deaths, etc.) and economic indicators (GDP, Health Expenditure per Capita, etc.) used in many studies (7, 15–17). As can be seen, the variables that measure the effectiveness of the health system examined in the literature generally have annual data. The annual data review does not allow a short-term strategy to contain such a pandemic period. In the non-annual studies conducted for the COVID period, a specific date range was taken, and comparisons were made between countries. In country comparisons made within a specific date range, since countries are caught in waves at different times, simultaneity cannot be obtained, and this makes it difficult for countries to compare their efficiency (1, 18–20). Our methodological approach preserves sample homogeneity when considering the abovementioned methodologies.

However, the point where these studies have the most difficulty and cannot reach the data is the absence of daily health data or the fact that daily health expenditures affect the data later and show effectiveness in these data in the long term. One of the significant problem encountered in the studies conducted to examine the COVID-19 period and its effects arises as being faced with a data set that is very difficult to measure in monetary terms. Moreover, the measurement of economic losses and indirect health expenditures due to daily closures also seems problematic. It is impossible to reach health expenditure data due to the implicit nature of many health expenditures, at least within the framework of the COVID-19 period. In order to overcome these measurement limitations, the hidden information in the number of daily cases of the COVID-19 was utilized in the study. If the number of cases can be brought under control or reduced, a structure emerges that we can call health expenditures effective or ineffective. The stability or controllability of data can be checked from the stochastic properties of that data. If the data we are interested in is stationary, that means that the data is under control. Data that is under control can be predicted for the long term. If the long-term can be predicted, then health expenditures can be changed accordingly, and efficiency can be increased. This chain of actions can show different dynamics in each wave and the phase of each wave. If covariance stationarity can be achieved in each wave's phase, then the case numbers in that case are under control. We can express this more formally as follows:

**Lemma 1**. Let *y*_*t*_ be the number of cases per day. The number of cases per day is an indicator of health expenditure.

**Proof**


$$\begin{array}{l} HE=f\left(S,V,...\right) \end{array}$$

Health expenditure depends on many variables (stringency: *S* and vaccine: *V*). The same variables are also function of the number of COVID-19 cases *C* = *f*(*S, V*, ...). As we know from the SIR models, the contamination coefficient R, especially *R* = *f*(*S, V*), is a function of these two variables. As the stringency *S* and vaccine *V* increase, the contamination coefficient *R* decreases. We have come to the point where we can only show the daily number of cases from the transmission or contamination coefficients. In addition to this, health expenditure also contains the same data in its functional structure with a positive relationship, contrary to the number of cases. Since the relationship is as follows *HE* = *f*(*R*) ↗→ *C* = *f*(*R*) ↘, health expenditure efficiency can be detected following this relationship. Hence, decreasing number of daily cases indicate the health expenditure efficiency.

**Proposition 1**. Let *y*_*t*_ is the daily COVID-19 cases where *y*_*t*_ satisfies these conditions *y*_*t*_→*E*(*y*_*t*_) = μ , $E\left(y_{t}^{2}\right)=\sigma^{2}$, and *E*(*y*_*t*−*s*_, *y*_*t*−*j*_) = σ, s≠j → hence, this condition provides the health expenditure efficiency independent of other conditions.

**Proof**

It has shown that the *C* = *f*(*R*) in Lemma 1. The contamination rate R is calculated from the two consecutive day, hence, $R≅f\left(\frac{y_{t}-y_{t-1}}{y_{t-1}}\right)$. If this ratio is 1, $\frac{y_{t}-y_{t-1}}{y_{t-1}}=1$, then one person contaminated only one another person. If this ratio decreases then the contaminated one person contaminated less than one person and vice versa. Let us consider the contamination coefficient α for equal and less than case $\frac{y_{t}-y_{t-1}}{y_{t-1}}\leq \alpha$ . Now multiply both side of the inequality with *y*_*t*−1_; $y{/}_{t-1}\frac{y_{t}-y_{t-1}}{y{/}_{t-1}}\leq \alpha y_{t-1}$. And more over including the stochastic error term to this deterministic relation, we obtained this equation *y*_*t*_−*y*_*t*−1_ ≤ α*y*_*t*−1_+*u*_*t*_. With some algebra, we can obtain this form Δ*y*_*t*_ ≤ α*y*_*t*−1_+*u*_*t*_ which is very well-known Dickey and Fuller unit root test (21). If the ADF test result showed that the null hypothesis was rejected or the alternative hypothesis accepted α ≤ 0 then this means that *y*_*t*_ satisfies the following conditions, *y*_*t*_→*E*(*y*_*t*_) = μ , $E\left(y_{t}^{2}\right)=\sigma^{2}$, and *E*(*y*_*t*−*s*_, *y*_*t*−*j*_) = σ, s≠j. By using this proof for Proposition 1 and the proof from Lemma 1 showing that the stationarity of daily cases provides the health expenditure efficiency result.

**Corollary 1**. The conditions in Proposition 1 of daily case numbers with waves and phases can only be met by each phase or demanded series by cumulative Fourier function.

**Proof**

Let the cases estimated by the following function *y*_*t*_ = β_1_+α*y*_*t*−1_+*WP*_*t*_+*u*_*t*_→*WP*_*t*_ = ϕ(*t*). By using Fourier Representation Theorem that the cumulative Fourier functions estimated or approach to the wave and phases one to one $\phi \left(t\right)=\alpha_{0}+\overset{n}{\underset{i=1}{\sum }}\alpha_{i}\sin\left(\frac{2\pi kt}{T}\right)+\overset{n}{\underset{k=1}{\sum }}\beta_{k}\cos\left(\frac{2\pi kt}{T}\right)$. By using stochastic difference equation or simply a regression analysis we can find the best approximating n value for the cumulative Fourier transform where the n shows the number of cumulating. By using residual sum of square value $u_{t}^{2}=f\left(\beta \right)$ where **β** = (α_0_, α_1_, ..., α_*n*_, β_1_, β_2_, ..., β_*n*_) we find the best fitting n. Ordinary least square (OLS) **β** = (***X***′***X***)^**−1**^***X***′***Y*** is the optimization algorithm for finding the $\underset{\beta }{\min}\left(u_{t}^{2}\right)$ $y_{t}^{*}=y_{t}-\alpha_{0}+\overset{n}{\underset{i=1}{\sum }}\alpha_{i}\sin\left(\frac{2\pi kt}{T}\right)+\overset{n}{\underset{k=1}{\sum }}\beta_{k}\cos\left(\frac{2\pi kt}{T}\right)\to y_{t}^{*}=u_{t}$. Shortly we can demonstrate by this equation $y_{t}^{*}=\alpha^{*}y_{t-1}^{*}+u_{t}^{*}$. Hence the condition in Proposition 1 is satisfied. The condition now is satisfied with demeaned data, fortunately we can also divide the sample in to phase and wave by using the cumulative Fourier function and hence the conditions are also satisfied for each wave's phase as well. When we take the first derivative with respect to time and equating it to zero $\frac{d\phi \left(t\right)}{dt}=0$ we will obtain the optimum points of cumulative Fourier trend. The condition which we know from differential equation can be obtained by difference equation as follows: $\Delta \phi \left(t\right)=\alpha_{0}+\overset{n}{\underset{i=1}{\sum }}\alpha_{i}\sin\left(\frac{2\pi kt}{T}\right)+\overset{n}{\underset{k=1}{\sum }}\beta_{k}\cos\left(\frac{2\pi kt}{T}\right)-\alpha_{0}+\overset{n}{\underset{i=1}{\sum }}\alpha_{i}\sin\left(\frac{2\pi k\left(t-1\right)}{T}\right)+\overset{n}{\underset{k=1}{\sum }}\beta_{k}\cos\left(\frac{2\pi k\left(t-1\right)}{T}\right)=0$. In a more compact form $\Delta \phi \left(t\right)=\left(\overset{n}{\underset{i=1}{\sum }}\alpha_{i}\left(\sin_{t}-\sin_{t-1}\right)\right)+\left(\overset{n}{\underset{i=1}{\sum }}\beta_{i}\left(\cos_{t}-\cos_{t-1}\right)\right)=0$. Taking the second derivative with respect to time then will give the inflection points $\frac{d^{2}\phi \left(t\right)}{dt^{2}}=0$. These inflection points are helping us to divide the sample into phases. Therefore, the first derivative will give the peak points of wave and from the second derivative we will find the phases. Thus, we satisfied the condition which we obtained in Proposition 1 for the phases as well.

#### 2.2.1. Technical remark

Similar types of efficiency studies are also found in the finance literature. The most well-known of these is the efficient market hypothesis. The efficient market hypothesis says that the markets are unpredictable and that investors will not provide returns above the index's return. ADF test is used again to test this hypothesis, and it is tested that the null hypothesis shows the efficient market hypothesis, that is, that the series diverges and is unpredictable. On the contrary, in the alternative hypothesis, the market will be predictable, and above-index returns can be achieved. While the understanding of effectiveness comes from the unpredictability of the series here, predictability in the structure we propose shows the effectiveness of health expenditure: Δ*P*_*t*_ = α*P*_*t*−1_+*u*_*t*_. The null hypothesis applied to the equation leads to the result of market efficiency, while the alternative hypothesis leads us to the result of market inefficient; *H*_0_:α = 0 *H*_*a*_:α≠0.

### 2.3. Econometric modeling

Recent studies by Becker et al. (22), Enders and Lee (21, 23), Rodriques and Taylor (24), and others have used Flexible Fourier Transforms to represent smooth breaks. The Fourier approach has several benefits, such as being able to capture the behavior of a deterministic function of unknown form even if the function itself is not periodic, performing better than dummy variable methods whether the breaks are instantaneous or smooth, and not having to worry about choosing the dates, number, and type of breaks (21–24). All of these papers made the point that to avoid the over-filtration issue; the structural break assessment should be done using the single frequency component of the Fourier Transforms. Becker et al. (22) used the Fractional Frequency Flexible Fourier Form (FFFFF) for the Trig-test, a structural break test. They try to demonstrate why their approach is superior to the widely used break tests.

Moreover, the newly proposed Omay (25) test follows Becker et al. (22) and Enders and Lee (21) and combines their methodologies to obtain the FFFFF ADF test. However, our study uses cumulative frequency to investigate wave and phase-dependent unit root testing, which is a deviation from the previous studies. The previous studies concentrate on the single frequency to determine the smooth break, but we are searching for the wave and phase of the data-generating process.

For this purpose, we are using **Corollary 1** to introduce a new constraint on cumulative frequency. Therefore, this new constraint lets us determine the exact number of the cumulative frequency apart from Enders and Lee (21) and Omay (25). These studies assume the cumulative frequency to be a maximum of 5. However, we introduce a new condition for obtaining the correct timing of waves and phases. This new methodology enables us to find the correct number of cumulative frequencies and hence proper testing of the data, which covers wave and phase-dependent data-generating processes. As we know from the previous literature, the upper limit of cumulative frequency determination is not possible due to the goodness of fit measure of the residual sum of squares getting better and better with the increasing number of cumulative frequencies. Thus, it is impossible to stop increasing the cumulative frequency at a reasonable number of frequencies. Finally, we solve this problem for this specific data-generating process by using **Corollary 1**.

The following Dickey–Fuller test is considered;


(1) $$\begin{array}{l} y_{t}=d\left(t\right)+\phi_{1}y_{t-1}+\lambda t+\varepsilon_{t} \end{array}$$

in this equation, ε_*t*_ is a stationary disturbance with variance, while is a deterministic function of *t*. Omay (25) assume that the initial value is fixed, and ε_*t*_ has weak dependence, similar to Enders and Lee (21, 23). According to Enders and Lee (21, 23), if the functional form of *d*(*t*) is known, it is feasible to estimate Eq. (1) and evaluate the null hypothesis of a unit root. Any test for is difficult if *d*(*t*) is misidentified when the form of *d*(*t*) is unknown. Omay (25) test and Enders and Lee (21, 23) tests are predicated on the notion that by using the Fourier expansion, one can approximate by *d*(*t*):


(2) $$\begin{array}{l} d\left(t\right)=\alpha_{0}+\alpha_{}\sin\left(\frac{2\pi kt}{T}\right)+\beta_{k}\cos\left(\frac{2\pi kt}{T}\right) \end{array}$$

where *T* is the total number of observations and k is a specific frequency. When there is no non-linear trend, all α_*k*_ = β_*k*_ = 0 values result in the DF test, a specific case of the test. Use of a large number of cumulative frequencies is unsuitable for a variety of reasons. Specific frequency *k* = 1 is frequently a good approximation to a model with structural change, as advised in the literature. However, we concentrate on the cumulative frequency and estimate the n for the best-fitting wave and phase-dependent non-linearity. Therefore, using Corollary 1 leads us to obtain the sharp type of change in the data correctly. Until now, we have explained all the details of Fourier type of unit root testing. Nevertheless, from now on, we are considering only Corollary 1 to proceed in the empirical part.


$$\begin{array}{l} \Delta y_{t}=\rho y_{t-1}+c_{1}+c_{2}t+\overset{n}{\underset{i=1}{\sum }}c_{3,i}\sin\left(\frac{2\pi kt}{T}\right) \end{array}$$

(3) $$\begin{array}{l} +\overset{n}{\underset{i=1}{\sum }}c_{4,i}\cos\left(\frac{2\pi kt}{T}\right)+e_{t} \end{array}$$

Now we can proceed with economic intuition behind the testing equation. With the number of daily cases, regardless of the health investments of the countries, the pandemic process is indirectly determined by the number of cases. In a similar study, Mulligan (26) and Barasa et al. (3) assumed that in the presence of infectious disease, the costs of infection were proportional to the number of infected people and stated that they were proportional to the number of interactions, that is, to the transmission coefficient. They control the dramatic results of the epidemic with the number of new cases per day measured depending on the number of tests valid for both the transmission stage and the diagnosis and treatment stage. Their study revealed that countries prepare for such a crisis differently regarding the organization and leadership of the health system (27). These differences have also caused differences in the pandemic process of countries. Therefore, this affects the wave and phase lengths and the contamination coefficients. Based on these studies and our Proposition 1, the behavior of the pandemic process was examined in two stages. Due to the lack of monthly and daily data on health expenditure, the indirect method proposed in Section 2.2 is used. The number of daily cases in the first stage was divided into waves and phases according to the contamination coefficient with the Cumulative Fourier function. In the second stage, COVID-19 cases' stationarity is examined to investigate whether the countries make effective health expenditures according to waves and phases. The study aims to help countries make effective short- and long-term decisions. The research presented an opinion on the effectiveness of health expenditures indirectly over the number of daily cases in 5 OECD countries during the COVID-19 Pandemic. The sample start date was chosen as each country's first case notification day.

## 3. Empirical study and discussion

In the first stage of the study, the new daily cases taken until November in the pandemic process, which started with the detection of the first cases in February 2020, were estimated with the cumulative Fourier function. As can be seen from Table 2, Australia and Germany had three waves, while Canada, France, and America had four waves. While Canada, Germany, and France experienced the pandemic process with similar movements in almost the same period, Australia separated from these countries after the Second Wave. After the First Wave, all other countries except America were in the pivotal region where the number of cases (not fluctuating) remained stable until the beginning of the 2^nd^ Wave. It has an interim period of about two months, which we can describe as, throughout the pilot region, countries were able to keep the number of cases stable. While Australia and America completed their second Wave in the 3^rd^ quarter of 2020, other countries experienced the 2^nd^ Wave until the 1^st^ quarter of 2021. Australia managed to keep the number of cases at a reasonable level for about 10 months after the 2^nd^ Wave. With this analysis, it has been shown that countries experience different conditions of the pandemic at different times. A similar study; is the study of Al-Saidi et al. (27), who found that countries respond differently to the pandemic process due to different readiness.

**Table 2:** The estimation of wave and phases by using cumulative Fourier transform.

		**AUS**		**CAN**		**DEU**		**FRA**		**USA**	
		**Date range**		**Date range**		**Date range**		**Date range**		**Date range**	
Wave 1	Phase 1	26.1.20	6.3.20	26.1.20	18.3.20	27.1.20	7.3.20	24.1.20	20.3.20	22.1.20	17.3.20
	Phase 2	7.3.20	26.3.20	19.3.20	20.4.20	8.3.20	28.3.20	21.3.20	10.4.20	18.3.20	13.4.20
	Phase 3	27.3.20	10.4.20	21.4.20	27.5.20	29.3.20	12.4.20	11.4.20	29.4.20	14.4.20	30.4.20
	Phase 4	11.4.20	1.5.20	28.5.20	30.6.20	13.4.20	3.5.20	30.4.20	21.5.20	1.5.20	21.5.20
Wave 2	Phase 1	18.6.20	10.7.20	21.8.20	21.11.20	10.9.20	12.10.20	18.7.20	11.10.20	22.5.20	19.6.20
	Phase 2	11.7.20	31.7.20	22.11.20	25.12.20	13.10.20	13.12.20	12.10.20	5.11.20	20.6.20	27.7.20
	Phase 3	1.8.20	19.8.20	26.12.20	1.2.21	14.12.20	16.1.21	6.11.20	22.11.20	28.7.20	25.8.20
	Phase 4	20.8.20	14.9.20	2.2.21	28.2.21	17.1.21	22.2.21	23.11.20	15.12.20	26.8.20	17.9.20
Wave 3	Phase 1	5.7.21	10.9.21	1.3.21	27.3.21	23.2.21	24.3.21	16.12.20	10.3.21	18.9.20	10.11.20
	Phase 2	11.9.21	6.10.21	28.3.21	21.4.21	25.3.21	16.4.21	11.3.21	1.4.21	11.11.20	26.12.20
	Phase 3	7.10.21	2.11.21	22.4.21	16.5.21	17.4.21	12.5.21	2.4.21	24.4.21	27.12.20	30.1.21
	Phase 4			17.5.21	9.7.21	13.5.21	16.6.21	25.4.21	22.6.21	31.1.21	9.3.21
Wave 4	Phase 1			10.7.21	21.8.21	23.7.21	4.11.21	23.6.21	17.7.21	18.6.21	2.8.21
	Phase 2			22.8.21	16.9.21			18.7.21	10.8.21	3.8.21	1.9.21
	Phase 3			17.9.21	5.10.21			11.8.21	31.8.21	2.9.21	25.9.21
	Phase 4			6.10.21	3.11.21			1.9.21	30.9.21	26.9.21	22.10.21

Dates are given as day, month, year.

For this reason, to compare the effectiveness of their countries' health systems, they are examined by dividing them into waves and phases according to the contagion coefficient. The predictability of the number of cases means that the cases are under control and the process is managed effectively. Therefore, stationarity means that health expenditures are also carried out effectively. The related cumulative Fourier estimation results can be seen below in Table 2:

In the second stage of the study, the unit root test was applied to investigate the stationarity of the phases. The results show waves and phases where case numbers are stationary. According to the results obtained by using the intercept and trend model (W1P1), it was determined that the cases could not be predicted, especially in the first phase of the pandemic (Table 3) (Australia, Canada, Germany, and the US). In contrast, France, whose case numbers seemed stationary in the first days of the pandemic, lost control of the cases in Phase 2. Phase 3 and Phase 4 were stationary in general of the waves. This situation can be interpreted as France's policy being different from other countries against a possible pandemic shock. In the relief phase and the second wave, it is seen that the countries that were severely affected at the beginning of the epidemic started to control the number of cases by taking adequate measures, thus increasing the efficiency of their health systems. Similar results were obtained by Lupu and Tiganasu (1). The common feature of all the countries we examined is that phase 1, which represents the beginning of the waves, is not stationary. This result may mean that the onset of waves is unpredictable. It was observed that Germany and US could not manage the process consistently. However, until the Omicron variant, Germany and Australia had three pandemic waves, while Canada, France, and the US had four pandemic waves. Hence, this situation may be due to the different case management and, therefore, the health policy implementation of the countries. As can be seen, from the unit root test results in Table 3 of the cases, it can be claimed that predictability of the number of cases for each wave and phase, so they cannot make effective health expenditures. All these results confirmed our Lemma 1, Proposition 1, and Corollary 1. Therefore, if the daily COVID-19 cases are stationary, the countries will reach efficiency at that phase.

**Table 3:** The unit root test results showing the efficiency of health expenditure.

	**AUS**		**CAN**		**DEU**		**FRA**		**USA**	
	**Intercept**	**Intercept & trend**	**Intercept**	**Intercept & trend**	**Intercept**	**Intercept & trend**	**Intercept**	**Intercept & trend**	**Intercept**	**Intercept & trend**
W1P1	−0,514	0,917	4,181	3,983	4,386	6,067	−8,472^***^	−8,056^***^	1,649	2,503
W1P2	−0,206	−4,028^**^	−2,109	−3,481^*^	−0,324	−3,108	−3,484^**^	−3,210	−1,858	−0,811
W1P3	−1,875	−5,170^***^	−1,980	−5,040^***^	−2,752^*^	−4,271^**^	−5,266^***^	−4,691^**^	−3,909^**^	−3,572^*^
W1P4	−4,725^***^	−6,074^***^	−2,177	−3,212^*^	−2,160	−6,267^***^	−3,691^**^	−3,887^**^	−4,154^***^	−3,133
W2P1	0,881	−3,459^*^	0,111	−2,936	6,085	3,635	2,178	−1,211	2,425	1,633
W2P2	−0,281	−3,964^**^	−5,810^***^	−6,042^***^	−2,374	−1,971	−3,060^**^	−5,187^***^	−1,933	1,348
W2P3	−0,364	−5,522^***^	−0,811	−4,392^***^	−4,969^***^	−5,183^***^	−2,531	−5,676^***^	0,069	−4,414^***^
W2P4	−1,934	−4,322^**^	−5,404^***^	−5,906^***^	−2,005	1,290	−4,503^***^	−5,187^***^	−3,337^**^	−3,156
W3P1	1,923	−0,653	2,494	0,595	2,318	−4,135^**^	−1,410	−2,089	4,172	0,876
W3P2	−1,269	−4,137^**^	−4,206^***^	−5,183^***^	−1,315	−4,487^**^	−3,490^**^	−5,854^***^	−4,017^***^	−4,702^***^
W3P3	3,071	−2,754	−3,813^***^	−5,480^***^	−3,606^**^	−5,746^***^	−6,505^***^	−6,311^***^	−0,715	−2,070
W3P4			−3,196^**^	−1,754	−1,679	−5,283^***^	−1,847	−4,330^***^	−1,844	−1,596
W4P1			2,873	−1,096	2,033	0,839	0,339	−3,681^**^	8,384	1,306
W4P2			−5,835^***^	−4,642^***^			−3,882^***^	−4,967^***^	−1,535	−3,029
W4P3			−4,946^***^	−4,787^***^			−1,878	−6,469^***^	−4,644^***^	−4,446^**^
W4P4			−5,796^***^	−5,245^***^			−2,017	−0,634	−0,555	−1,831

*, **, *** are representing the %10, %5, and %1 significance level. W and P representing wave and phase.

## 4. Conclusion and recommendation

The study analyzed the effectiveness of public health spending in 5 OECD countries during the COVID-19 Pandemic. The results showed that countries experienced the pandemic process of varying lengths and intensities and that dividing the daily number of cases into waves and stages according to the contagion coefficient is a more accurate method. The unit root test performed in the second stage showed that health expenditures were effective in waves and phases where the number of cases was stationary. The results naturally showed that the onset of waves was unpredictable, and countries could not make effective health expenditures for each wave and stage. In the study, it was concluded that the periods when countries made effective health expenditures during the pandemic period were when they started to control the number of cases by taking adequate precautions after being seriously affected by the epidemic.

The findings of this study have important implications for public policies in managing pandemics. Firstly, the results highlight the importance of preparedness and effective health systems. In all the first phases of the countries, the daily COVID cases are found unit root process, which indicates that they are not prepared at the first phases and lead to inefficiency in their health expenditure. The unpredictability of the pandemic's first phase in most countries highlights the need for countries to have a robust plan to manage the pandemic effectively. Secondly, the study results show that the countries experience the pandemic process differently and at different times, highlighting the importance of a tailored and flexible approach to public health policy. Each country should design its policies based on its specific situation and needs. Thirdly, examining health expenditures indirectly through daily data allows for examining the capacity and ability of national economies worldwide to prevent, detect, and respond rapidly to the emergence of infectious diseases and other acute forms of public health endangerment. Finally, the results show the importance of data analysis in pandemic management. It is possible to get an idea about the trends and patterns in the spread of the pandemic by applying the Cumulative Fourier function and the unit root test, which we employ in the study. This information can be used to inform public policies and make evidence-based decisions.

Of course, the effectiveness of health expenditures alone does not stop or slow the pandemic. Health expenditures will be effective when people's cultural behaviors and the level of democracy of countries are taken together with stringency measures, which are called non-pharmaceutical measures. In future studies, the effectiveness of the measures taken in the pandemic process can be measured by examining short-term data on the pandemic process and these variables together. Thus, the decisions to be taken by policymakers can be improved.

## 5. Limitation of the study

Like all studies, this study also has certain limitations that should be considered.

Firstly, the study is limited by the data used. The analysis is based on the number of daily cases; some cases may have been missed or underreported. Therefore, the correctness of the data limitation could lead to inaccuracies in the results and conclusions.

Secondly, the study only focuses on five advanced countries (Australia, Canada, France, Germany, and America), and it is possible that the results may not be generalizable to other countries. The experiences of these countries may not be representative of the experiences of other countries. Further studies would be needed to examine the impact of the pandemic on different countries.

In conclusion, the limitations of this study should be considered when interpreting the results and conclusions. Further studies would be needed to address these limitations and to gain a more comprehensive understanding of the impact of the pandemic on different countries.

## Data availability statement

The datasets presented in this study can be found in online repositories. The names of the repository/repositories and accession number(s) can be found below: https://ourworldindata.org/covid-cases.

## Author contributions

EB: conceptualization, investigation, review and editing, resources, data curation, writing, formal analysis, visualization, and writing—original draft preparation. KBA: conceptualization, investigation, review and editing, and supervision. TO: conceptualization, methodology, formal analysis, investigation, resources, and writing. All authors contributed to the article and approved the submitted version.
